# The Phenomenology of Bereavement: Sensory Experiences of the Deceased

**DOI:** 10.1192/j.eurpsy.2025.752

**Published:** 2025-08-26

**Authors:** A. F. Silva, A. C. Rodrigues, A. C. Matias-Martins, F. Santos, P. Coelho, R. M. Lopes, T. Vieira, V. Melo

**Affiliations:** 1 Psiquiatria, Unidade Local de Saúde do Médio Tejo, Tomar, Portugal

## Abstract

**Introduction:**

Many grieving individuals report sensory and phenomenological experiences involving deceased loved ones, such as visual, auditory, or sensed presences. These experiences are common across cultures, with 40-70% of bereaved spouses reporting them. They can provide comfort or cause distress, and can last from brief moments to several years. Despite their prevalence, there is limited empirical research on these occurrences, leaving mental health professionals often unprepared to address them.

**Objectives:**

This study aims to explore the phenomenology of sensory experiences with the deceased, the factors associated with these experiences, and their impact on grieving individuals.

**Methods:**

A bibliographic review was conducted using PubMed, using terms like “Bereavement,” “Hallucinations,” “Phenomenology,” and “Sensory Experiences.”

**Results:**

The review indicates that sensory experiences involving deceased loved ones should not automatically be viewed as pathological. Rather, these experiences may function as adaptive responses that help maintain emotional bonds with the deceased, facilitating the grieving process. Research highlights that “sensing a presence” is the most frequently reported phenomenon, followed by visual and auditory encounters. Key factors influencing these experiences include sensory deficits, cognitive difficulties and poor sleep.These phenomena are particularly common among older adults, with women more likely than men to report them. Strong emotional ties, marital satisfaction, and having children with the deceased correlate positively with these sensory experiences. High levels of avoidant coping are significant predictors of experiencing these phenomena, while a more detached coping style appears to help in accepting the finality of the loss. Studies suggest that those who have these experiences often report greater levels of prolonged grief, PTSD, depression, and feelings of emotional loneliness compared to those who do not. However, these experiences do not necessarily result in better or worse clinical outcomes. Mental health professionals must create a supportive environment where individuals can discuss these experiences without fear of judgment or being labeled as mentally ill. Understanding these phenomena as psychologically meaningful and significant to the bereaved can promote healing.

**Conclusions:**

This review emphasizes the need for a nuanced understanding of sensory experiences involving the deceased, moving beyond simplistic interpretations as signs of mental illness. Instead, these experiences should be seen as contextually meaningful and part of the bereaved’s relationship with the deceased. For those distressed by these experiences, therapeutic interventions could focus on reshaping their relationship with the deceased or modifying their responses.

**Disclosure of Interest:**

None Declared

